# *Ex-vivo *HRMAS of adult brain tumours: metabolite quantification and assignment of tumour biomarkers

**DOI:** 10.1186/1476-4598-9-66

**Published:** 2010-03-23

**Authors:** Alan J Wright, Greg A Fellows, John R Griffiths, M Wilson, B Anthony Bell, Franklyn A Howe

**Affiliations:** 1 Cardiac and Vascular Sciences, St George's, University of London, London, UK; 2 Academic Neurosurgery Unit, St George's, University of London, London, UK; 3 Cancer Research UK Cambridge Research Institute, Cambridge, UK; 4 Cancer Sciences, University of Birmingham, Birmingham, UK; 5 Birmingham Children's Hospital NHS Foundation Trust, Birmingham, UK

## Abstract

**Background:**

High-resolution magic angle spinning (HRMAS) NMR spectroscopy allows detailed metabolic analysis of whole biopsy samples for investigating tumour biology and tumour classification. Accurate biochemical assignment of small molecule metabolites that are "NMR visible" will improve our interpretation of HRMAS data and the translation of NMR tumour biomarkers to *in-vivo *studies.

**Results:**

1D and 2D ^1^H HRMAS NMR was used to determine that 29 small molecule metabolites, along with 8 macromolecule signals, account for the majority of the HRMAS spectrum of the main types of brain tumour (astrocytoma grade II, grade III gliomas, glioblastomas, metastases, meningiomas and also lymphomas). Differences in concentration of 20 of these metabolites were statistically significant between these brain tumour types. During the course of an extended 2D data acquisition the HRMAS technique itself affects sample analysis: glycine, glutathione and glycerophosphocholine all showed small concentration changes; analysis of the sample after HRMAS indicated structural damage that may affect subsequent histopathological analysis.

**Conclusions:**

A number of small molecule metabolites have been identified as potential biomarkers of tumour type that may enable development of more selective *in-vivo *^1^H NMR acquisition methods for diagnosis and prognosis of brain tumours.

## Background

Nuclear Magnetic Resonance (NMR) Spectroscopy has been used to assign and quantify the small molecule metabolites in brain tumours. *In-vivo *Magnetic Resonance Spectroscopy (MRS, reviewed in [1]) allows for the assignment and quantification of small molecule metabolites in intracranial tumours. The various intracranial tumour types have different metabolic profiles in ^1^H MR spectra and pattern recognition of these profiles has been used to develop a decision-support system that can assist in the radiological diagnosis and grading of brain tumours [2]. The main potential of MRS in radiological diagnosis lies in aiding binary decisions between tumour types that can be challenging for a radiological diagnosis using conventional MRI [3]; for example, between lymphoma and glioblastoma (GBM) or metastasis and GBM. While a "black-box" pattern recognition approach may prove useful for such diagnostic decisions it is also important to have a biological understanding of the spectra, particularly in the assignment and quantification of individual metabolites, as this may allow refinement of the acquisition protocol towards improving the accuracy of tumour classification. The low spectral resolution of the current *in-vivo *MRS in clinical practice produces spectra dominated by the larger peaks (e.g. Creatines, Cholines, myo-Inositol (m-Ins), lipids and macromolecules) that mask the contribution of smaller resonances -e.g. taurine (Tau) and glycine (Gly)- to the overall profile. As spectral resolution improves, due to higher field strength MRI systems becoming more readily available (e.g. 3 T for routine clinical use and up to 9.4 T for clinical research), *in-vivo *MRS will be able to detect increasing numbers of metabolites. The quantification of these metabolites may provide useful biomarkers for diagnosis and prognosis of brain tumours. *Ex-vivo *^1^H- High-Resolution Magic Angle Spinning (HRMAS) NMR provides quantitative information on these small molecule metabolites in unprocessed brain tissue [4], but at a much higher spectral resolution than is currently available in clinical MRS studies, providing an indication of all the NMR-visible metabolites that are potentially observable *in vivo*. Additionally the tissue sample is available for further analysis (e.g. histopathology or genetic) after the experiment. HRMAS has been previously used in several studies of brain tumour tissue [5-11] and the concentrations of the main tumour metabolites that are measured show good agreement between *in-vivo *MRS and *ex-vivo *HRMAS for several tumour types [10,12-14]. Other pathologies have also been investigated using quantitative *ex-vivo *HRMAS [15,16]. This paper provides assignments for metabolites that are observable for the most common histopathological types of adult intracranial tumour (gliomas of grade II to IV, meningiomas, metastases and also lymphomas) and reports their quantitation. Metastatses are the most common intracranial tumours [17] while GBM, other gliomas, meningiomas and lymphomas account for 17%, 13%, 30% and 3% respectively of diagnoses of primary CNS tumours in the USA [18]. The assignment and quantification presented here provides sets of metabolites that may prove significant in future *in-vivo *MRS studies of various adult-intracranial tumours. This paper extends previous HRMAS studies of intracranial tumours, which have for example, identified the common metabolites in normal brain [19], in some individual adult [7,9] and pediatric [13] brain tumours. We now extend this work to include more brain tumour types (lymphoma and metastases), with additional small molecule metabolites assigned and quantified. Novel assignments of small molecule metabolites in particular brain tumour types have also been made that may provide biomarkers to aid specific tumour classifications. This paper also extends quantification of brain tumour metabolites in gliomas [8] to a range of adult intracranial tumours. An evaluation of changes in metabolite concentration that may occur as a result of the experimental conditions and the effects of tissue damage on histopathological re-examination post-HRMAS are also included in this work. Concentration measurements were made using LCModel [20] software that fits the spectral data with a linear combination of spectra of individual metabolites. The basis set of these metabolite spectra are modelled using computer simulation [21] based on the chemical shift assignment determined in this current work.

## Results and Discussion

### Peak assignments, metabolite concentrations and artefacts

#### Assignment of NMR visible small molecule metabolites

**Figure 1:**
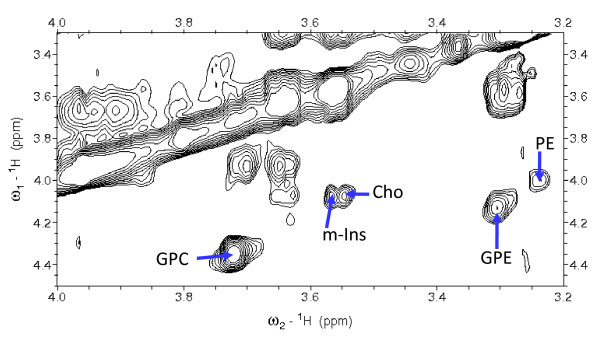
**TOCSY showing GPE cross peak**. A region of a TOCSY spectrum from a GBM sample showing the cross peak assignment of GPE. Cross peaks are indicated for selected metabolites; the GPE resonance is at 3.3 ppm in the direct dimension and 4.1 ppm in the indirect dimension.

Analysis of a subset of 15 samples of five different histopathological types of brain tumour allowed for the assignment of 29 small molecule metabolites. These metabolites are acetate (Ace), alanine (Ala), ascorbate (Asc), aspartate (Asp), betaine (Bet), choline (Cho), creatine (Cr), glucose (Glc), glutamate (Glu), glutamine (Gln), glutathione (GSH), glycerophosphocholine (GPC), glycerophosphoethanolamine (GPE), glycine, histidine (His), hypotaurine (h-Tau), isoleucine (Ile), lactate (Lac), leucine (Leu), myo-inositol, N-acetyl-aspartate (NAA), phosphocholine (PCh), phosphoethanolamine (PE), scyllo-inositol (s-Ins), succinate (Suc), taurine, threonine (Thr) and valine (Val). CPMG (Carr-Purcell-Meiboom-Gill) spectra, with a total echo time of 50 ms were collected to measure metabolites chemical shift. Macromolecules are larger than the small molecule metabolites and as a result they have significantly quicker T2 relaxation. The CPMG sequence acts as a T2 filter, greatly reducing the signal of these faster relaxing macromolecules compared to the smaller metabolites. There are smaller differences in T2 relaxation between the metabolites leading to variations in their peak heights, widths and resolution from neighbouring peaks. These variations make the quantification from CPMG spectra unreliable and so they are only used for assignment. Initial assignments of previously reported metabolites were made with reference to the literature, as listed in Table 1, with the main sources of reference being Govindaraju et al. [19] and Martínez-Bisbal et al. [7]. Assignments for most metabolites (Ace, Ala, Asc, Asp, Cho, Cr, Glc, Glu, Gln, GSH, GPC, Gly, Ile, Lac, Leu, Lys, m-Ins, NAA, PCh, s-Ins, Suc, Tau, Thr, Val) are well established in the literature of brain tumours [6,22-25] other tumours [26] and tumour cell culture [27] with *in-vitro *and *ex-vivo *high resolution NMR. However, the primary references of Govindaraju et al[19] and Martínez-Bisbal et al. [7] are peak assignments from solution NMR of common brain metabolites and of resonances specific to GBM respectively. Further, novel assignments have been made for metabolites previously unreported in some of these tumour types; for example, His and Bet have been observed and assigned in some glioma spectra in this study. GPE assignments were made using TOCSY (Total Correlation Spectroscopy) spectra as their peaks overlap with bigger resonances in one dimensional spectra. GPE has a clearly defined cross peak in some spectra (see Figure 1) that was assigned with reference to its presence in HRMAS spectra of prostate [28] and cultured melanoma cell lines [29]. H-Tau has been reported in meningiomas [9,24] and in astroglial [30] and glioma cell lines [27,31] and we have now observed this *ex vivo *in gliomas and lymphomas as well as meningiomas. Asc is unreported to date in NMR spectra of brain tumours and here was assigned with reference to the Human Metabolomics Project database [32,33], the Biological Magnetic Resonance Bank small molecule database [34] and a 1D pulse acquire spectrum acquired from a 50 mM Asc solution with 10% D_2_O and 10 mM sodium formate as a chemical shift reference.

**Table 1:** Chemical shift assignments of specific metabolites

Metabolite	Group	Chemical Shift (ppm)
Asc	CH	4.521

	CH	4.019

	CH_2_	3.752

		3.729

		

Bet	CH_2_	**3.889**

	N(CH_3_)_3_	3.271

		

GPE	CH_2_	4.110

	CH_2_	**3.959**

		**3.856**

	CH	**3.882**

	CH_2_	**3.681**

		**3.618**

	CH_2_	3.278

		

His	CH	3.961

	CH_2_	3.144

		3.087

		

H-Tau	CH_2_	3.344

	CH_2_	2.647

This table shows the chemical shift assignments of selected metabolites that have not been previously reported in particular types of brain tumour. The assignments are referenced to the methyl resonance of Cr at 3.03 ppm. Generally, assignments were made with reference to CPMG and/or TOCSY spectra of 15 samples representing 5 different types of tumour. Assignments in bold are taken from the literature assignments [19,33].

Other, relatively small, resonances still remain unassigned. A singlet was observed at 3.69 ppm, present in seven spectra of varying histopathological type, and a multiplet at 2.2 ppm was observed in 2 astrocytoma II spectra. A doublet at 1.16 ppm was observed in five meningioma and 2 GBM's CPMG spectra that is tentatively assigned as the methyl group of 3-hydroxybutyrate with reference to previous studies [24,31,35]. However, although cross peaks are expected in the TOCSY at 2.4 and 3.5 ppm for 3-hydroxybutyrate no 2.4 ppm cross peak was observed in the relevant samples. This may be due to a sub-optimal mixing time in the 2D TOCSY for observing this resonance or the typically low concentration of this metabolite. Either way the assignment cannot be confidently confirmed.

#### Chemical Shift variation of small molecule metabolites

Variation in the chemical shift of peaks between HRMAS experiments have been noted previously [21], the most likely cause of which is that pH, salt content and the ratio of D_2_O to H_2_O are parameters that are not controllable in the same way as, for example, NMR of metabolite solutions obtained from acid extractions of tissue. If there is any variation in the flow of air to the rotor then there is the possibility of subtle temperature variations resulting in small chemical shift variations. It is also possible that there is a systematic variation in chemical shift between tumour types due to consistent differences in the metabolite environments associated with a type of tumour. In this regard, differences in the pH and chemical environments within cells and tissues of tumours may vary the chemical shifts of some metabolites. To ascertain the extent of this variation the chemical shift of 7 peaks that were observable in each of 7 meningioma, 7 GBM and 7 metastases spectra were measured with the CH_3 _resonance of Cr set to 3.03 ppm as a reference. The list of the peaks, their line width at half maximum height and standard deviation of their chemical shift are given in Table 2. There is a small, but non zero, variation in the 3.93 ppm Cr singlet (standard deviation 0.39 Hz) which may be due to inaccuracy introduced by the sampling rate, variations in the shim or even changes in relative size of overlapping signals in the 3.9 ppm region. Similar variations in the resonance position of the selected metabolites are observed. However, this variation is small compared to the resonance peaks' line width-at-half-height which are 4 to 11 times greater than their positional standard deviation. The separation between the Lac CH and CH_3 _signals varied by 0.69 Hz, a similar value to the variation in the separation of the two Cr singlets. It seems that variation in peak separation is similar to that of peak position relative to the Cr reference and much smaller than linewidth. Individual t-tests of the chemical shift variation of each peak in Table 2 show that there are no significant differences (p ≤ 0.05) between tumour types. This result validates the use of a single basis set of simulated spectra (see below) to be used in LCModel to fit the spectra of all the different tumour types.

**Table 2:** Chemical shift variation in 15 CPMG spectra

Resonance	Ala doublet 1^st ^peak	Cr CH_2 _singlet	Gly singlet	Gln multiplet 3^rd ^peak	Lac doublet 1^st ^peak	PCh singlet	Tau triplet 2^nd ^peak
Chemical shift (ppm)	1.48	3.93	3.55	2.44	1.34	3.27	3.42

Standard deviation of chemical shift in (Hz)	0.19	0.39	0.55	0.29	0.32	0.37	0.69

Average line width at half height of peak (Hz)	2.3	3.0	2.3	3.5	2.3	3.3	2.5

This table shows the average chemical shift for 7 peaks from 15 CPMG spectra that have been referenced to the CH_3 _resonance of Cr. The standard deviation of these mean chemical shifts and the average line widths of the peaks are also given.

#### Quantification of metabolites using LCModel

**Figure 2:**
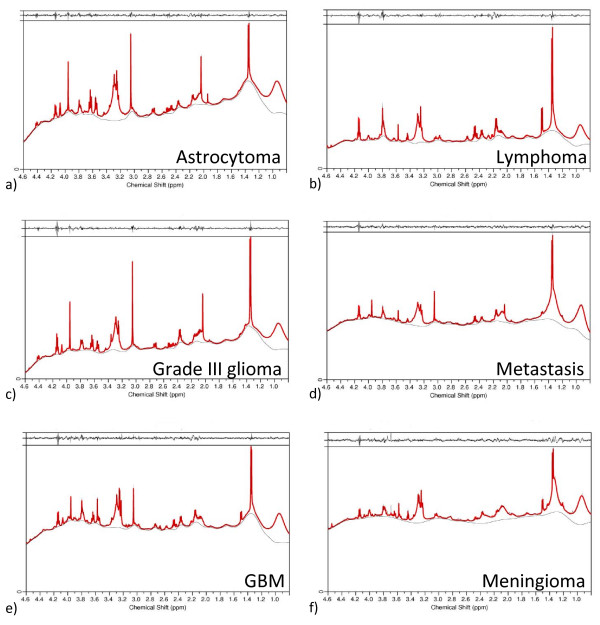
**LCModel fits of HRMAS spectra**. LCModel was used to fit the simulated basis set to each HRMAS pulse acquire spectrum with water presaturation. The six examples given here are a) astrocytoma grade II, b) lymphoma, c) an anaplastic astrocytoma, d) metastasis, e) GBM and f) a meningioma. The red line shows the linear combination of the basis set, and the thin grey line is the base line determined by LCModel. The residual (the fit and the base line subtracted from the raw data) is shown in a narrow box at the top of each panel.

Individual spectra were quantified for small molecule metabolites using a simulated basis-set of metabolite spectra and LCModel. A pulse-acquire spectrum was acquired for determining metabolite concentrations and a second (unsuppressed) spectrum was acquired to measure the amount of H_2_O in the sample as a reference signal. The mean concentration and its standard error for metabolites are given for each tumour type in Table 3. Examples of LCModel fits to spectra with their residuals are given in Figure 2 for different histopathological types of tumour. The residuals are generally small compared to the metabolite signals. The Cramér-Rao lower bounds are on average 34% for all metabolites, with an average value of less than 25% for 15 of the metabolites (Ala, Bet, Cho, Cr, Gln, Glu, Gly, GPC, GSH, Lac, Leu, Lys, m-Ins, PCh, Tau). Overall 71% of all metabolite fits in all of the 65 spectra have a Cramér-Rao lower bound less than 25%. Lac, which is ubiquitous and at high concentrations, has just a 3% Cramér-Rao lower bound on average. In practice LCModel may completely miss a low level metabolite peak thus giving it an artifactual zero concentration. Reviewing the CPMG and TOCSY data showed that this occurred for only 38 assignments of the 29 metabolites evaluated over 65 spectra (i.e. 2%).

**Table 3:** Mean metabolite concentrations by tumour group

	Ast II	Grade III	GBM	Metastases	Meningioma	Lymphoma
Ace Ace	*0.17 ± 0.10*	*0.22 ± 0.06*	*0.19 ± 0.04*	*0.15 ± 0.02*	*0.18 ± 0.03*	0.40 ± 0.03

Ala	*0.30 ± 0.13*	1.06 ± 0.31	1.32 ± 0.17	0.81 ± 0.17	**2.75 ± 0.46**	2.59 ± 1.06

Asc	0.46 ± 0.08	0.58 ± 0.09	0.63 ± 0.10	*0.60 ± 0.28*	0.82 ± 0.14	**2.94 ± 1.41**

Asp	0.41 ± 0.13	*0.37 ± 0.43*	*1.14 ± 0.33*	*0.18 ± 0.12*	*0.19 ± 0.06*	*0.73 ± 0.37*

Bet	0.14 ± 0.03	0.17 ± 0.06	0.25 ± 0.04	0.10 ± 0.02	0.16 ± 0.03	0.21 ± 0.08

Cho	0.24 ± 0.05	0.26 ± 0.07	0.33 ± 0.05	0.23 ± 0.04	0.23 ± 0.04	0.34 ± 0.03

Cr	3.31 ± 0.80	3.07 ± 1.35	2.99 ± 0.39	1.24 ± 0.21	0.65 ± 0.13	2.07 ± 1.39

Glc	0.53 ± 0.22	*0.19 ± 0.13*	0.38 ± 0.12	*0.64 ± 0.31*	*0.12 ± 0.07*	0.55 ± 0.55

Glu	1.51 ± 0.60	3.01 ± 1.39	2.60 ± 0.33	2.28 ± 0.46	**4.94 ± 0.56**	4.46 ± 0.42

Gln	5.12 ± 0.56	4.63 ± 1.35	7.11 ± 0.89	2.13 ± 0.32	**7.54 ± 0.92**	5.44 ± 1.18

GSH	0.52 ± 0.15	0.55 ± 0.11	0.93 ± 0.11	0.96 ± 0.30	2.04 ± 0.25	1.18 ± 0.07

GPC	**1.21 ± 0.14**	0.77 ± 0.21	0.76 ± 0.14	0.73 ± 0.32	0.26 ± 0.07	0.42 ± 0.15

GPE	0.62 ± 0.13	1.20 ± 0.58	*0.41 ± 0.10*	0.69 ± 0.15	1.03 ± 0.27	*0.60 ± 0.09*

Gly	1.59 ± 0.54	1.52 ± 0.47	2.34 ± 0.39	0.75 ± 0.11	1.51 ± 0.22	2.18 ± 0.49

His	0.58 ± 0.13	0.65 ± 0.47	**1.67 ± 0.53**	*0.08 ± 0.06*	*0.09 ± 0.04*	0.82 ± 0.82

h-Tau	0.35 ± 0.12	*0.58 ± 0.21*	**1.16 ± 0.23**	*0.06 ± 0.03*	0.69 ± 0.20	0.36 ± 0.04

Ile	*0.13 ± 0.02*	*0.15 ± 0.04*	*0.11 ± 0.02*	*0.25 ± 0.06*	*0.26 ± 0.06*	*0.46 ± 0.24*

Lac	5.68 ± 0.66	8.91 ± 1.56	9.04 ± 1.07	5.40 ± 0.71	9.81 ± 0.89	8.83 ± 0.76

Leu	0.26 ± 0.03	0.92 ± 0.46	0.51 ± 0.09	0.56 ± 0.10	0.62 ± 0.11	1.14 ± 0.45

Lys	0.52 ± 0.20	0.61 ± 0.12	1.07 ± 0.12	1.05 ± 0.22	1.29 ± 0.17	2.31 ± 0.85

m-Ins	**5.23 ± 0.92**	*2.02 ± 0.83*	2.03 ± 0.32	*0.70 ± 0.23*	*0.30 ± 0.08*	*1.36 ± 0.40*

NAA	1.00 ± 0.65	1.14 ± 0.76	0.48 ± 0.15	*0.29 ± 0.11*	*0.07 ± 0.02*	1.08 ± 1.07

PCh	0.36 ± 0.12	0.45 ± 0.07	**1.56 ± 0.27**	0.68 ± 0.11	0.97 ± 0.12	1.10 ± 0.20

PE	*0.18 ± 0.05*	1.14 ± 0.76	1.97 ± 0.30	1.93 ± 0.30	1.87 ± 0.27	**4.55 ± 1.72**

s-Ins	0.25 ± 0.05	0.20 ± 0.10	0.20 ± 0.03	*0.10 ± 0.03*	*0.04 ± 0.01*	0.26 ± 0.04

Suc	*0.11 ± 0.02*	*0.13 ± 0.04*	0.19 ± 0.03	*0.14 ± 0.03*	0.28 ± 0.04	*0.18 ± 0.02*

Tau	1.00 ± 0.14	1.00 ± 0.23	0.85 ± 0.11	1.81 ± 0.77	1.75 ± 0.25	2.25 ± 0.74

Thr	0.31 ± 0.06	0.43 ± 0.16	0.66 ± 0.29	0.29 ± 0.07	0.44 ± 0.13	0.90 ± 0.18

Val	0.20 ± 0.04	0.31 ± 0.11	0.20 ± 0.04	0.37 ± 0.04	0.38 ± 0.05	**1.02 ± 0.45**

The metabolite concentrations of 65 quantified tumour samples grouped into general tumour types (Astrocytoma II, glioma grade III, GBM, metastasis and meningioma). Metabolite concentrations are given (in mM) as group arithmetic means ± the standard error of this mean. Relatively high metabolite concentrations that are referred to in the text as specific markers for particular tumour types are highlighted in bold type. Metabolites, for each tumour type, with a median Cramér-Rao lower-bound of the peak fitting that is greater than 25% of the peak volume, are given in italics.

The good quality fits observed in Figure 2 were achieved with a limited set of simulated macromolecules given in Table 4. Each resonance in Table 4 is a Lorentzian singlet generated by the LCModel software to simulate (and fit) a broad macromolecule peak. Fewer macromolecule peaks are used in the LCModel fits presented here than in previous LCModel analyses of *ex-vivo *tissue [36,37]. The macromolecule set used here was determined as the minimal set required to achieve flat residuals in LCModel quantifications, with no unfitted broad resonances in any of the quantified spectra. With only one peak simulated for lipid resonances at 1.33 ppm (which vary in frequency with the distance of the CH_2 _group from the terminal methyl of the lipid) it is possible that this peak, and other lipids and macromolecules, may not be completely fitted in the analysis. This is compensated for by the allowed variability in these simulated peaks (see Table 4) and by the variability in the base line that LCModel fits to the data. The spline base line fitted in LCModel often includes part of the broad resonance signals (as can be seen for the 1.3 ppm resonance in Figure 2, panels a, b, c and e) thus making the quantification of macromolecules unreliable. For semi-quantitative measurements of these peaks, more detailed simulated macromolecule basis sets are required [36] though this is not the focus of this paper.

**Table 4:** Macromolecule peaks simulated in LCModel

Molecular group of the resonance	Chemical shift (ppm)	Line width (Hz)
Lipid -C**H**_3_	0.969 ± 0.05	0.1 ± 0.09

Lipid -C**H**_2_-...-C**H**_2_-C**H**_2_-CH_3_	1.333 ± 0.05	0.1 ± 0.09

Lipid -C**H**_2_-CH_2_-COOH	1.609 ± 0.01	0.04 ± 0.02

Lipid -C**H**_2_-CH =	2.056 ± 0.01	0.04 ± 0.02

Lipid -C**H**_2_-COOH	2.277 ± 0.01	0.04 ± 0.02

Lipid = CH-C**H**_2_-CH =	2.829 ± 0.01	0.04 ± 0.02

Lipid = CH-C**H**_2_-CH =	2.858 ± 0.01	0.04 ± 0.02

MM (protein)	3.285 ± 0.01	0.04 ± 0.02

The simulation parameters of broad macromolecule resonances for LCModel that were included in the fit of each spectral quantification. The chemical shift and line width with their allowed range of variability (±) are given alongside the molecular group that each broad singlet is simulating (highlighted in bold). MM (protein) refers to a variety of protein signals that produce a broad resonance in this region.

**Figure 3:**
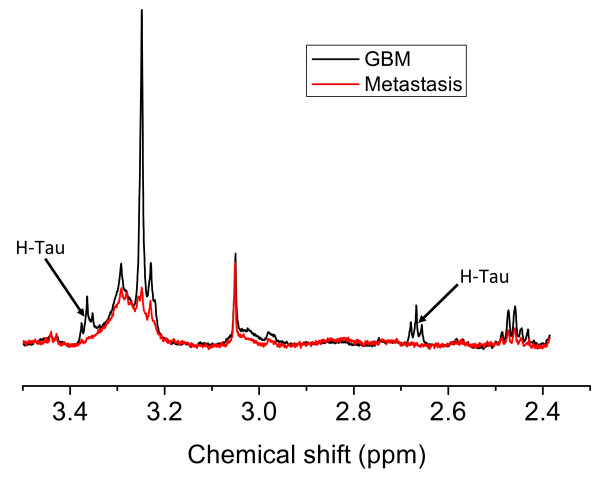
**Absence of h-Tau in metastases**. This figure shows two pulse-acquire specta. The spectrum of a GBM shows the position of the two triplet resonances of h-Tau. The metastasis shows noise in these same regions of the spectrum despite LCModel fitting a small amount of h-Tau when quantifying this spectrum.

Table 3 indicates those metabolites, within a particular tumour type, that have high errors of fitting in grey. The metabolites have a median value of the Cramér-Rao lower-bound greater than 25%. One example, as can be seen in Table 3, is h-Tau in metastases. Metastases have an LCModel-calculated average concentration of 0.06 mM of h-Tau; however this is an overestimation as there are no clearly observable h-Tau resonances in the eight metastases samples. This highlights a natural bias in LCModel in that metabolites are included if it results in an improvement in the fit even if it does so without much confidence. The uncertainty is reflected in the Cramér-Rao lower bound estimates that range between 45% and 495% for h-Tau in metastases, with a median value of 125%. An example is given in Figure 3 of a region of a metastasis spectrum that had 0.07 mM calculated h-Tau concentration, yet there is only noise where the expected h-Tau resonances would be, as observed in the GBM spectrum. A similar result is found for His, which although not observable in any of the metastasis or meningioma spectra, are given small average concentrations as measured by LCModel of 0.08 mM and 0.09 mM respectively. The importance of using the Cramér-Rao lower bound to assess the reliability of a concentration measurement is highlighted by these measurements.

#### Changes in metabolite concentration during the experiment

**Figure 4:**
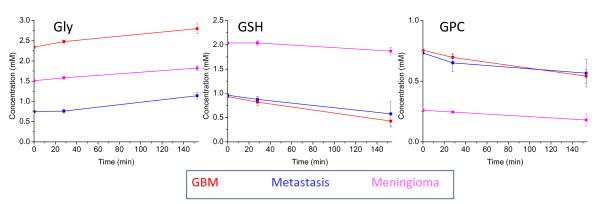
**Metabolite concentration changes over time under HRMAS experimental conditions**. These three line graphs plot the change in mean metabolite concentration with time over the course of 28 minutes and 2 hours 33 minutes. Error bars show the standard error of the change in the mean. Changes are shown for Gly, GSH and GPC which have significant changes over 2 hours 33 minutes and the different tumour types in each plot are indicated by the colour key.

T-tests were performed on 87 metabolite average concentration changes over 2.5 hours to see if there were statistically significant deviations from zero, with p < 0.01. There was a significant drop in the concentration of GPC and GSH in GBMs and a rise in Gly in all three tumour types over three hours. These three significant changes in GBMs over 2.5 hours are consistent with a previous GBM study [38] that found these changes, under similar experimental conditions (3 hr, 5000 Hz spinning at 4°C), with Gly being the largest change in both studies (an average increase of 0.46 mM in this study and 0.34 mM previously [38]). A previous GBM study [38] also reported rises in Ace, Lac, Leu and Cho and a fall in m-Ins; these metabolites changed similarly in this current study though not significantly. The increase in Cr, Glu and Ala previously observed [38] were not observed in this current study, but instead a non-significant decrease occurred. The reasons for the greater number of significant changes in the previous study may be the slightly longer time course (3 hours as opposed to ~2.5 hr in our study). Changes over 28 minutes and 2.5 hours are shown for Gly, GSH and GPC in Figure 4. The graphs suggest that for the three different tumour types there is a fall in GPC and GSH and a rise in Gly. These changes are of the order of less than 0.5 mM over three hours so are not significant in the case of simply acquiring the spectra for measuring metabolite concentration, which can be performed with 15 minutes of data acquisition and within half an hour from the start of sample spinning at 4°C. One possible exception is Gly that has a significant (p = 0.007) rise in concentration over 28 minutes in GBMs. This suggests that Gly is at a higher 'NMR visible' concentration in an HRMAS sample than in the *in vivo *tumour. The source of this extra Gly could be metabolic processes or the release of a 'bound' species due to sample damage that occurs in the HRMAS experiment. Gly could be released by the action of proteolytic enzymes on cellular proteins in the sample. In contrast, most metabolites have not changed significantly in metastases, meningioma and GBM though each has slight losses of GSH and GPC. It is possible that the GPC and GSH concentrations given here are lower when compared to tumour concentrations *in vivo *though any changes in the time taken to acquire quantitative 1D spectra will be small. GSH and GPC concentration changes are similar for three different histopathological types of tumour, therefore any comparisons in relative concentration of these metabolites between tumour types (see below) should still be valid.

Our current measurements can indicate the size of metabolite changes observed during conditions of an HRMAS experiment but not of those that occur prior to that point. Sample ischaemia is known to produce rapid increases in Ala and Lac concentrations (reviewed in [38]) such that these metabolites (along with Glc) are significantly different between *in-vivo *and *ex-vivo *measurement [14]. These metabolite concentrations will be artificially high in *ex-vivo *studies, but that does not necessarily suggest that the results presented here for these metabolites are not without significance [13]. Ala has been found to be significantly higher in meningiomas compared to other tumour types in this *ex-vivo *data (see the section on "Statistical analysis of individual metabolite differences between tumour types") which is in good agreement with its observation in significant concentrations in meningiomas *in vivo *[39,40]. Rapid increases in Ala is also a feature of the early stages of epileptic seizures prior to the formation of neuronal lesions [16].

#### Histopathology of post HRMAS samples

**Figure 5:**
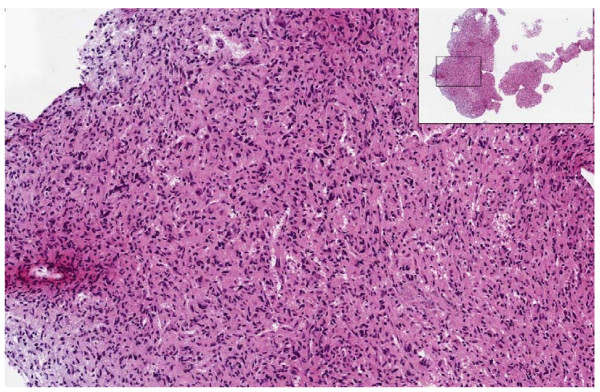
**Post-HRMAS histopathology of a GBM sample**. Hematoxylin and eosin stained slide of a fixed, post-HRMAS sample from a GBM patient. The insert shows the entire cut section on the slide with the characteristic fragmenting of the sample that occurs during the HRMAS experiment. The main picture shows a high density of disorganised nuclei indicative of tumour but not the features of high grade malignancy that would confirm a diagnosis of GBM.

After the HRMAS protocol 52 samples were fixed in formalin, stained with hematoxylin and eosin and sent for histopathological review for comparison with the initial histological diagnosis. A single GBM sample had deteriorated so much during the HRMAS that there was no sizeable portion of intact tissue left to fix. Other samples fractured into multiple pieces on removal from the rotor but were still fixed and stained successfully (see the insert panel of Figure 5 for an example). Table 5 summarises the results of post HRMAS histopathology in which the histopathologist was asked to assess the tissue for damage, necrosis and to make a most likely diagnoses of these 52 intact samples. The same tumour type (glioma, metastases, meningioma or lymphoma) was identified for 38 samples (73%) in post HRMAS histopathology compared to the original diagnosis. Of these agreements in tumour type, 10 glioma samples had different grade of malignancy compared to the original diagnosis. Table 5 shows GBM grade of HRMAS sample was lower than the grade determined from the full clinical diagnosis in all but 2 cases, with grade II (and in one case grade III) astrocytoma being suggested instead. An example of a GBM sample graded as astrocytoma II post HRMAS is given in Figure 5 showing a lack of malignant features that would confirm a high grade diagnosis. A previous study [5] suggested that post-HRMAS, GBM samples sometimes lack characteristic malignant and necrotic histopathological features and that regions of necrosis are compressed by sample spinning. The underestimation of glioma grade may be the result of avoiding necrotic regions when preparing the sample or changes due to sample spinning.

**Table 5:** Post-HRMAS histopathological review of samples

Clinical diagnosis	Number reviewed	Matching histology	Normal tissue	Necrotic or damaged	Mismatched histology
Ast. II	4	3	1	0	

Grade III	5	3	1	1	1 Ast. II

GBM	12	2	0	1	8 Ast. II 1 Grade III

Metastasis	8	6	1	0	1 Meningioma

Meng.	20	11	0	4	4 Glioma 1 Ependymoma

Lymphoma	3	3	0	0	

This table summarises the histopathological diagnoses of the tumour samples that were fixed and stained. Tumours are grouped as ast. II (astrocytoma II), grade III (the grade III gliomas), meng. (meningioma), metastasis and lymphoma. The table counts those that had matching diagnoses clinically and in post-HRMAS review and gives details where there was a mismatch. Those that were extensively normal appearing brain tissue and those too necrotic or damaged for histopathological verification are counted for each tumour group.

Six samples were too necrotic or damaged for post-HRMAS histopathology and there were five misdiagnoses (four meningiomas and one metastasis) which all had very small and/or highly damaged samples that severely compromised the ability to make a robust histopathological diagnosis. There were also three samples (two astrocytomas and one metastasis) where brain tissue but no tumour cells were observed. These samples had elevated levels of NAA which is a marker for normal brain (glial and neuronal cells), with 2 to 4 mM in the two glioma samples but only 0.8 mM in the metastasis; these concentrations are far lower than would be expected for a sample purely from normal brain [19]. The presence of normal brain cells highlights that in resected-diffuse astrocytoma or metastasis there is often some normal tissue excised with the tumour which may be sampled in the HRMAS experiment. This tissue may be structurally more robust and so dominate the post HRMAS sample for histopathological analysis.

Discrepancies in tumour grade and undiagnosable samples are the result of damage to the sample in a minority of post-HRMAS histopathological reviews. Damage to the samples can be greatly reduced with shorter experiment times than are used here, which include the 2D TOCSY experiments used for detailed resonance assignment. With shorter protocols there would be fewer samples lost due to mechanical damage from spinning. Slower spinning speeds may also reduce sample damage and could be employed as long as spinning side bands remain outside the metabolite regions of the spectra. Even in the examples presented here, with 2.5 hours spinning at 5 kHz, a majority of samples still had large numbers of observable tumour cells and a correct diagnosis in post-HRMAS histopathology. However, we do not recommend that such long HRMAS times are used if correlation to detailed histopathological analysis post-HRMAS is a major aim.

### Statistical Analysis and determination of tumour biomarkers

#### Statistical analysis of individual metabolite differences between tumour types

Significant differences in metabolite concentrations between histopathological types were found for Ala, Asp, Cr, Glu, Gln, GSH, GPC, Gly, His, h-Tau, Ile, Lac, Lys, m-Ins, NAA, PCh, PE, s-Ins, Tau, Val using a Kruskal-Wallis test of the concentration data. Individual Mann-Whitney-U tests were performed for each of these metabolites to compare their concentrations pair-wise between histopathological tumour types (summarised in Table 6). These results show a variety of differences at a high level of significance (p < 0.01) between glioma, lymphomas, metastases and meningiomas. These metabolites have potential as biomarkers for tumour differentiation in these binary comparisons. Significant differences were not observed between the groups with five or less samples (lymphoma, astrocytoma II and grade III glioma) as there are too few repetitions to give significant results at p < 0.01. There are also no significant differences between grade III glioma and GBM which may be the result of, the heterogeneity of the grade III glioma group that contains astrocytomas and oligoastrocytomas, the small sample size of grade III glioma group, or the heterogeneity of grade IV gliomas. There is similar difficulty in separating grade III and IV gliomas *in vivo *by MRS. Meningiomas show the largest number of metabolite differences when compared to the other tumour types. In particular meningiomas have significantly lower Cr concentrations than lymphomas, grade II astrocytomas, GBMs and metastases and higher Ala and Glu than these groups. In *in-vivo *MRS at 1.5T, Ala and the broad peak of Glu and Gln (referred to as Glx and observable at 2.4 ppm) have been previously proposed as markers of meningiomas [39,40]. The data presented here suggests that Gln in meningiomas is at a similar concentration to GBM (see Table 3) but Glu is larger in meningiomas which may account for the increase in the combined Glx signal. GSH has been previously suggested as an *in-vivo *marker of meningiomas when compared to normal brain and gliomas [41] and is significantly higher compared to all three glioma grades in this current data.

**Table 6:** Significant differences in metabolite concentration in binary comparisons between tumour type

Tumour type 1	Tumour type 2	Significantly higher concentration in tumour type 1	Significantly higher concentration in tumour type 2
lymphoma	Astrocytoma II		

lymphoma	Astrocytoma III		

lymphoma	GBM	Tau*	

lymphoma	Metastasis	h-Tau	

lymphoma	Meningioma	Cr*	Ala**, Glu*, Gln**, Lac*

Astrocytoma II	Astrocytoma III		

Astrocytoma II	GBM	m-Ins*	Ala

Astrocytoma II	Metastasis	Gln*, m-Ins*,	PE*

Astrocytoma II	Meningioma	Cr**, GPC**, His**, m-Ins**, s-Ins**	Ala**, Glu*, GSH**, PE

Astrocytoma III	GBM		

Astrocytoma III	Metastasis	h-Tau*	

Astrocytoma III	Meningioma	GPC*, m-Ins*	GSH*, Lys

GBM	Metastasis	Cr, Gln*, Gly*, h-Tau*	

GBM	Meningioma	Asp, Cr**, GPC*, His*, m-Ins**, NAA*, s-Ins**	Ala, Glu*, GSH**, Ile*, Tau, Val*

Metastasis	Meningioma	Cr*	Ala**, Glu*, Gln**, Lac*

A non-parametric Mann-Whitney-U test was used to compare for significant differences in metabolite concentrations between two general tumour types. This was repeated for all binary comparisons of the tumour groups with a significance level of p < 0.01. Significantly higher tumours are listed by abbreviation. Higher significance levels are indicated with asterisks * = p ≤ 0.005, ** = p ≤ 0.001.

An important distinction clinically is between metastasis and glioblastomas [42], tumours which can have a similar radiological appearance [3]. The Mann-Whitney-U test suggests significant positive biomarkers for GBM would be Cr, Gly, Gln and h-Tau. Conversely there are no potential positive biomarkers for metastasis in this comparison.

There are significant differences in the levels of two metabolites between low grade gliomas and GBMs. Astrocytoma IIs have higher m-Ins and GBMs have higher Ala. These are two potential positive biomarkers of high and low grade glioma in agreement with other *ex-vivo *HRMAS work [6]. Furthermore, high m-Ins is a feature of *in-vivo *short echo time spectra of astrocytomas but not of GBMs [1,2,43]. Although studies by Kinoshita et al. [44,45] of acid extracts of brain tumour samples showed an increase in m-Ins and Ala with tumour grade, the discrepancy may be the result of using an acid extraction method as compared to whole tissue.

#### Multivariate analysis of metabolite differences between tumour types

**Figure 6:**
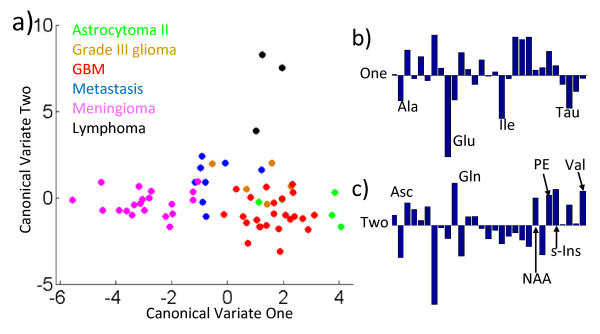
**MANOVA analysis of normalised metabolite concentrations**. a) Scatter plot of the first two canonical variables of the MANOVA analysis of the 6 different tumour types. Each individual sample is plotted with a colour particular to its tumour type as indicated in the key. b) The relative contributions of the normalised amount of metabolite that form Canonical Variate One are expressed as a bar chart. The most significant negative contributions in the linear combination are labelled with the metabolite abbreviation. c) The bar chart showing the combination that forms Canonical Variate Two. Here the largest positive contributing metabolites are labelled.

**Figure 7:**
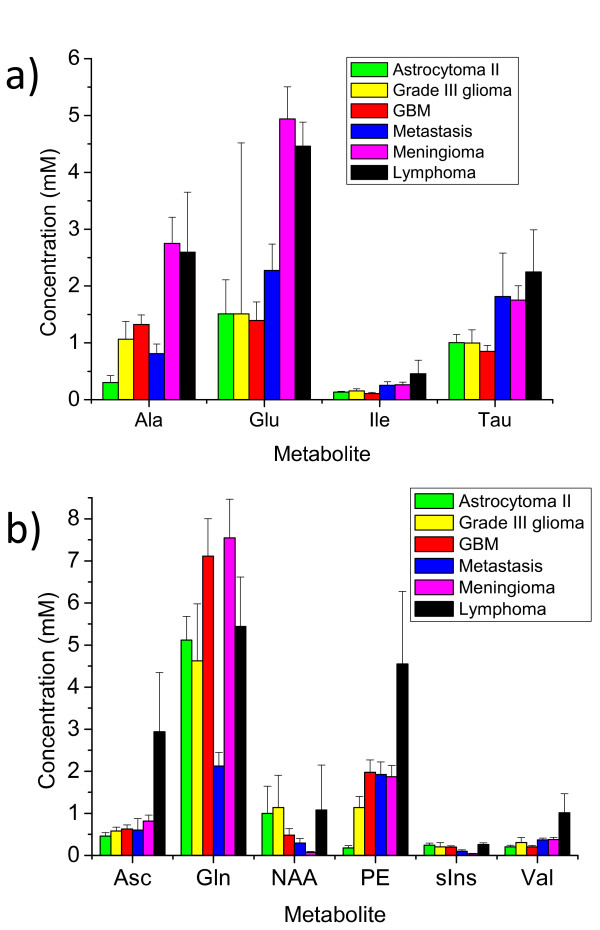
**Bar charts of key metabolites in MANOVA**. a) The mean metabolite concentrations (in mM) for four metabolites in each of the six groups of tumour indicated by the key. These metabolites contribute the most to Canonical variate 1 in Figure 6b. Error bars are given as the standard error of the mean. These are the four metabolites that were found to be important in the MANOVA separation of meningiomas from other tumour types. Due to the small number of samples the error in the estimation of Glu in Grade III gliomas makes any comparison statistically insignificant. b)The mean metabolite concentrations (in mM) for six metabolites in each of the six groups of tumour indicated by the key. These metabolites contribute the most to Canonical variate 1 in Figure 6c. Error bars are given as the standard error of the mean. These are the four metabolites that were found to be important in the MANOVA separation of lymphomas from other tumour types.

A multi-variate analysis of variance (MANOVA) has been performed on the tumour metabolite concentration data. This statistical test assumes a normal distribution of the data, which is generally not the case for metabolite concentrations. Despite this, the analysis can be used as a supervised clustering technique, also called discriminate analysis, by plotting canonical variables as in Figure 6. Canonical variables are linear combinations of the different amounts of each metabolite in each sample. The concentrations of metabolites are normalised by dividing each metabolite concentration by its mean over all 65 samples. The first canonical variate is calculated as the linear combination of the normalised metabolite-concentrations that produces the most significant analysis of variance (ANOVA) between the histopathological groupings. The variables of this first canonical vector are plotted on the *x *axis in Figure 6a. The second canonical variate (*y *axis) is the second most significant ANOVA in the data that is orthogonal to the first canonical variate. As can be seen from Figure 6a, the majority of the meningioma samples are separated from the other tumours types by having low values of canonical-variate 1. The relative contributions of the normalised metabolite concentrations to this variate are given in Figure 6b. Negative values of high magnitude that contribute to this variate include Glu and Ala. These have already been found to be significantly high in meningiomas compared to other tumour types. The other high contributing metabolites are Ile and Tau. Bar charts of these metabolites are given in Figure 7a. From the bar chart it can be seen that Ile and Tau may provide positive markers for meningiomas when compared to gliomas. Tau is also significantly higher in meningiomas than in GBMs (see Table 6). However Ile and Tau would not separate meningiomas from metastases and lymphomas, which have similar concentrations of these metabolites (Figure 7a).

Canonical variate 2 clearly separates the three lymphomas from the grouping of all the other tumour types, suggesting that there are characteristic metabolites that can be used for lymphoma classification. MRS biomarkers for lymphoma may prove useful as the radiological appearance of these tumours can sometimes resemble that of glioblastoma [46,47]. Strong positive contributions to the linear combination of canonical variate 2 are labelled in Figure 6c. Six metabolites (Asc, Gln, NAA, PE, s-Ins, Val) showed these strong positive contributions, of which, three: Asc, PE and Val show particularly high mean concentrations compared to other tumour types (see Figure 7b). These three metabolites may prove useful biomarkers of lymphomas in comparison to other common types of brain tumour. None of these three metabolites were significantly different between lymphoma and other tumour types in the Mann Whitney U analysis (see Table 6) though this is unsurprising as there were only three lymphoma samples in the data set. Asc is a novel assignment in this study and was found in most of the tumour samples with the highest average concentration in lymphomas. It is unclear as to the significance of its presence though. Asc is thought to provide an important anti-oxidant function in normal brain and Asc radicals are cytotoxic to glioblastoma cell lines [48]. Asc has been observed in normal brain with *in-vivo *MRS using the MEGA-PRESS spectral editing techniques [49]. Consideration of the known (prescribed) medication of patients at the time of surgical resection did not suggest any possible extrinsic sources of Asc.

#### Other differences in metabolite concentration between tumour type

**Figure 8:**
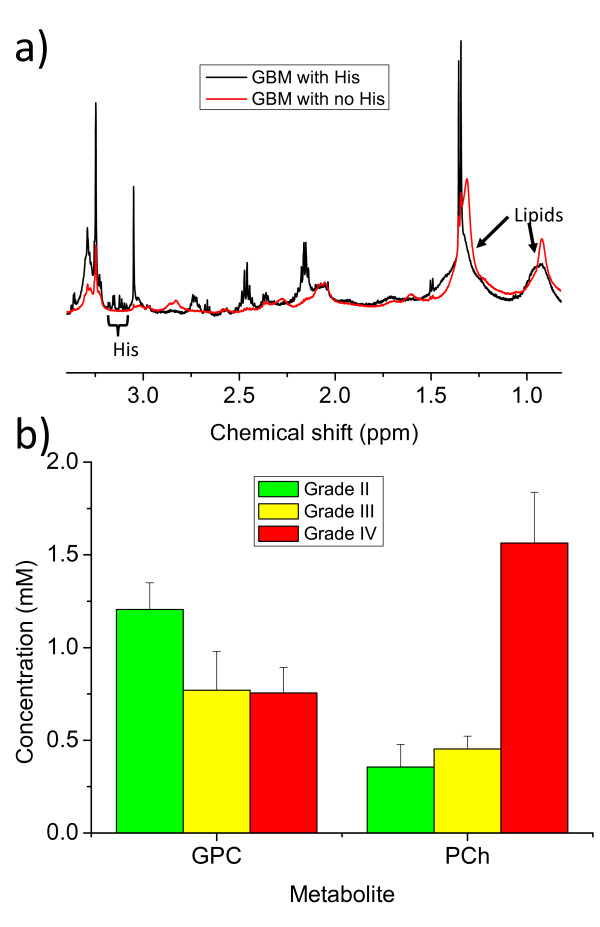
**Other metabolite differences between tumour types**. This figure shows two pulse acquire spectra of GBM samples. The spectrum shown in black has clear His signals in the 3.2 - 3.1 ppm region. The spectrum in red has only noise in this region and generally lower small molecule metabolite signals but much higher lipid signals as indicated in the 1.3 ppm and 0.9 ppm regions. b) This bar chart plots the mean concentration of GPC and PCh, with error bars of the standard error of the mean, for three of the tumour groups: astrocytoma grade II, grade III gliomas and the (grade IV) GBMs.

There are particular metabolites only observable in the spectra of some tumour types that could provide useful positive biomarker for tumour diagnosis. H-Tau is significantly higher in lymphomas, GBMs and astrocytoma grade III tumours than metastases. The absence of NMR-observable h-Tau from metastasis could make it a useful positive biomarker for GBM in a binary GBM-metastasis comparison. His could, similarly, be a useful positive biomarker for gliomas when compared to metastases. However, there is a complete absence of observable His in 10 of the 24 GBM samples, which may reflect the low metabolite concentration of these more necrotic GBMs, which have higher lipid signals as shown in Figure 8a. His has resonances in a spectral region (3.2 - 3.1 ppm) that do not overlap with other strong signals though other studies have reported tyrosine and phenylalanine [7] resonances in glioblastomas in this region. His is a possible target for a selective pulse sequence in *in-vivo *spectroscopy at field strengths higher than 3 Tesla but the low concentration, particularly in necrotic tissue, may make it undetectable in many *in-vivo *GBMs.

M-ins and Ala are significantly different between the low grade glioma and GBM in this study. Comparing average metabolite concentrations between the groups can give some insight into other potential markers of tumour grade. It has been previously reported that Grade II astrocytomas have higher GPC and lower PCh than grades II and IV [50]. The results presented here (see Figure 8b) suggest a similar trend of increasing PCh and decreasing GPC with grade, which was also observed previously in high resolution *in vitro *NMR of pechloric acid extractions of gliomas [51]. The relative amounts of GPC and PCh could prove useful markers of tumour grade in *in-vivo *MRS in the future if higher fields are employed to resolve their resonances.

## Conclusions

*Ex-vivo *HRMAS spectroscopy can reveal the NMR visible small molecule metabolites from adult brain tumours. Careful assignment of these metabolites has allowed novel assignments not previously reported for particular brain tumour types. These include the identification of Asc and GPE in various adult brain tumours, h-Tau in gliomas, lymphomas and meningiomas, and Bet in gliomas. The quantification of brain tumour metabolites has allowed the identification of significant differences in the amounts of particular metabolites, and these could provide novel biomarkers of brain tumour histopathological type *in vivo*. There are statistically significant higher concentrations of Cr, Gln and h-Tau in GBMs compared to metastases that may prove useful positive biomarkers for high-grade glioma versus metastasis, as may His, which is not observable in metastases. A MANOVA analysis revealed that lymphomas have a distinctive metabolic profile. Average concentrations for Asc, PE and Val are high for lymphomas though the low sample number (n = 3) requires further samples to be analysed to confirm these findings.

Concentration measurements have also identified various positive markers for different brain tumour types that correspond to previous *in-vivo *and *ex-vivo *studies [2,6,39-41,43,50]. In this regard meningiomas have significantly high Ala and low Cr compared to other metabolites. Glu has been shown to be the component of *in-vivo *"Glx" peaks that are high in meningiomas. There is also evidence to support m-Ins, GPC, Ala and PCh as biomarkers for discriminating low and high grade glioma.

Concentration measurement by HRMAS spectroscopy requires careful experimental design as Gly in glioblastomas increases significantly in the time period required to acquire quantitative 1D spectra. Furthermore, long HRMAS protocols (2.5 hr) at high spinning speeds (5 kHz) can cause tissue damage that compromises post HRMAS analysis in some samples, and particularly those that have necrotic regions. HRMAS has been used to confirm potential biomarkers for brain tumour prognosis and diagnosis currently identified from *in-vivo*, *in-vitro *and *ex-vivo *techniques. The extra resolution and analysis of unprocessed tissue that HRMAS provides has allowed novel identification of further biomarkers, with potential use in clinical practice.

## Methods

### Sample collection

Samples were collected from 65 brain tumour patients at St George's, University of London. Informed consent was provided by each patient and the study was approved by the local ethics committee (Local Ethics Reference: 06/Q0803/184). Surgically resected brain tumours had samples snap-frozen in liquid nitrogen for subsequent HRMAS and other samples sent for routine histopathology. Consensus diagnosis was made for each of the brain tumour patients, by clinicians at St Georges Hospital, according to local practice that included histopathological analysis of multiple samples other than those used in this HRMAS study. Included in this study were 4 diffuse astrocytomas, a gemistocytic astrocytoma, 3 anaplastic astrocytomas, 2 anaplastic oligoastrocytomas, 24 GBMs, 3 lymphomas, 8 metastases and 20 meningiomas. The frozen samples were stored at -80°C until used for HRMAS. Due to the small sample numbers available the analysis in this paper has been limited to broad groups of related histopatology: astrocytoma grade II, grade III gliomas (including the astrocytomas and oligoastrocytomas), GBM, the meningiomas and a group of the lymphomas.

### Sample preparation and HRMAS

Samples were dissected on dry ice to give a 5 - 25 mg fraction that was inserted into a chilled 50 μl HRMAS rotor insert containing 10 μl of frozen 10 mM Sodium Formate (as a chemical shift reference) in D_2_O. The insert was filled with more D_2_O, capped, and quickly transferred into an HRMAS rotor (Bruker Biospin, Coventry, UK) that had been cooled on ice. The rotor was transferred to the NMR spectrometer that had been pre-cooled to a sample temperature of 4°C. HRMAS experiments were performed using a Bruker Avance 600 MHz NMR spectrometer fitted with a 4 mm quadruple-tuned HRMAS probe (^1^H, ^2^H, ^13^C, ^31^P). The sample was maintained at a constant internal temperature of 4°C and a spin rate of 5000 Hz. The sample was left at these conditions for 15 minutes prior to acquisition to allow temperature equilibration. A standard protocol for the experiments was set up:

1) Presaturation pulse acquire with a repetition time (TR) of 3.14 s and a total time for acquisition (TTA) of 3 min 48 s.

2) Presaturation pulse acquire TR 9.14 s, TTA 1 min 50 s

3) Pulse acquire TR 3.14 s, TTA 10 min 23 s

4) CPMG TTA 11 min 46 s

5) Presaturation pulse acquire TR 3.14 s, TTA 3 min 48 s

6) TOCSY, TTA 2 hrs 1 min

7) Presaturation pulse acquire TR 3.14 s, TTA 3 min 48 s

The full acquisition protocol was acquired for 53 of the 65 samples and acquisitions 1 to 4 were acquired for the remaining 12. All the one dimensional sequences were acquired with a band width of 7184 Hz and 8192 complex points. Sequence 3 had a water suppression of 60dB, 64 scans. Sequence 2 was identical to 3 except that no water suppression pulse was applied to enable tumour water content to be used as a reference signal for metabolite quantification and there were only 8 scans. Sequences 1, 5 and 7 were identical with 64 scans, sequence 5 occurred 28 minutes after the initial acquisition and sequence 7 was 2 hours and 33 minutes later. The CPMG sequence was acquired with a 50 ms echo time with inter-echo spacing of 0.4 ms, 128 scans and a repetition time of 6.14 s. TOCSY spectra were acquired with water presaturation, 16 scans of 7184 Hz bandwidth and a 70 ms DIPSI-2 isotropic mixing sequence in the direct dimension with 256 increments in the indirect dimension. The entire acquisition protocol took 2 hrs 37 minutes to perform, after which the rotor was immediately removed from the spectrometer placed onto ice and the tissue fragment taken out and placed into a 4% formaldehyde solution.

### Post-HRMAS histopathology

Once fixed, tissue fragments were put into paraffin blocks for sectioning and then stained with hematoxylin and eosin for histopathological analysis. Histopathological slide preparation and photography was provided by the CRUK Cambridge Research Institue (CRI, Robinson Way, Cambridge, UK). Post HRMAS histopathological analysis was provided by Dr. Andrew Dean (Adenbrookes Hospital, Cambridge UK). Two non-adjacent slices from each biopsy sample were assessed for percentage necrosis, sample damage, and were given a most-likely histopathological diagnosis of tumour type where possible. Histopathological analysis was completed for 52 samples.

### Processing of HRMAS data

For assignment, all spectra were Fourier transformed using TOPSPIN (Bruker Biospin), referenced to the Sodium Formate singlet at 8.45 ppm. The chemical shift and line widths at half height for the singlets of Cr, Gly, PCh, were measured using TOPSPIN by fitting lorentzian peak shapes to the CPMG spectra. Line-widths were similarly found for the higher field peaks of the doublets of Lac and Ala and the chemical shifts of these resonances (plus the Lac quartet) were also determined. The chemical shifts are reported in Table 2 relative to Cr at 3.03 ppm.

A GBM and a metastasis spectrum contained very high level signals from mannitol and so were not included in the set of 65 analysed samples.

### Simulated basis set of small molecule metabolites

A subset of 15 brain tumour samples of varying histopathological type (3 lymphoma, 3 astrocytoma II, 3 GBM, 3 metastases and 3 meningiomas) were analysed for the chemical shift of assignable resonances in their CPMG spectra. Where there were more than 3 spectra in a tumour type then representative spectra had to be selected (particularly for GBM, meningioma and metastases). The selection criteria were to choose samples with the most resonances, clearly resolvable above the noise, in both the CPMG and TOCSY spectra. An average chemical shift was calculated from each peak in as many of the CPMG spectra in which the resonance was present and resolved. Multiplets without clearly resolved peaks in one dimensional spectra were assigned chemical shift values from average peak positions in TOCSY spectra. The multiplets assigned in this way were 3.79 ppm of Ala, 3.30 ppm and 4.13 ppm of GPE, 3.84 ppm and 2.22 ppm of GSH, 1.43 ppm and 4.37 ppm of Thr and 3.46 ppm and 3.68 ppm of Glu. Further multiplet resonances were undetectable above the noise in any spectrum and were therefore assigned, relative to known shifts, from H_2_O solution values in Govindaraju et al. [19] or the Human Metabolome Project [33]. Some of these 'literature assignments' are indicated in Table 1 by the grey colour of the chemical shift. Basis sets were simulated using TARQUIN [11,21] with scalar coupling constants taken from Govindaraju et al. [19]. Additional scalar coupling information was obtained from the Human Metabolome Project database [33] or, in the case of Asc, were estimated from a 1D spectra of a pure solution. The TARQUIN software was used with MATLAB [52] to simulate the metabolite basis sets that most accurately reproduced the average peak positions from the 15 samples. A singlet was also simulated for quantification of the water resonance in acquisition 2 of the NMR experiments. Individual simulated spectra were combined into basis sets in LCModel for quantification of spectra.

### Simulation of macromolecule signals

Assignments of broad signals from lipid and protein macromolecules were also made from the subset of 15 spectra. These signals were fit with a small set of broad Lorentzian singlets, generated by LCModel for each analysis, with varying chemical shift and line width as detailed in Table 4.

### Quantification of spectra

The raw time domain data from the pulse acquire experiment of sequence 3 in the acquisition protocol was loaded into LCModel and quantified with the simulated basis set and the simulated macromolecule signals. For metabolite concentrations the unsuppressed water signal (from sequence 2 of the acquisition protocol) was also quantified using the TARQUIN-simulated singlet in a basis-set. Metabolite concentrations were calculated according to the formula:

which gives the quantification as a concentration of the metabolite (in mM) as if it were dissolved in the tissue water in the sample rather than in the complete tissue volume. It is a potential concern that the D_2_O added to the rotor exchanges with water prior to and during sample collection thus artificially diluting the metabolites. This was assessed by preparing a sample containing just the 10 mM formate solution in D_2_O, waiting the same amount of time until acquisition as for a tumour sample then performing a water-unsuppressed pulse acquire measurement. The relative concentration of H_2_O in the D_2_O can then be calculated revealing that there is a negligible amount of H_2_O in the 10 - 20 μl of added D_2_O compared to the water content of 5 - 25 mg of tissue.

### Statistical analyses

The average chemical shift of 7 different metabolite peaks was compared for 3 groups of tumour type, meningioma, GBM and metastases, using individual t-tests at a significance level of p ≤ 0.05 to reject the null hypothesis of no difference between tumour types. A Kruskall-Wallis test was performed to find metabolite concentrations that showed significant differences between groups of different tumour type (grade II astrocytoma, grade III glioma, GBM, metastases and meningioma; p ≤ 0.05). Mann-Whitney-U tests were performed to determine the significant metabolite concentration differences with binary combinations of the different tumour types and with a conservative significance level of p < 0.01. Changes in metabolite concentrations during the HRMAS experiment were determined from acquisitions 1, 5 and 7 in the spectroscopy protocol given above. These three identical pulse acquire spectra were collected at the start of the protocol then 28 minutes later and 2 hrs 33 minutes after the start. Changes in concentration were followed for 8 samples in each of three tumour types (meniongioma, GBM and metastasis) by LCModel fitting of the spectra. This allowed any metabolite concentration changes to be measured over these time periods. Metabolite concentrations were excluded from the analysis if they were incorrectly missed out from the LCModel fits; therefore there were a maximum (but not necessarily) 8 concentration changes measured for each of the three tumour types. In this regard, there were, on average, 5.6 repeat measurements for each of the 29 metabolites in each tumour type. Concentration changes were calculated for the ~2.5 hour period by subtracting the quantified amounts of acquisition 1 from acquisition 7. The concentration change was calculated by assuming the initial quantity was that measured from acquisition 3 (as described in the section "quantification of spectra"). Mean changes in metabolite concentration changes over ~2.5 hours for each tumour type were calculated and t-tested against a null hypothesis of no change in metabolite concentration. Similar calculations were made for metabolite changes over the 28 minute period between acquisition 1 and 5. The Kruskall Wallis, Mann Whitney U, and t-tests were performed using SPSS [53].

MANOVA was performed using the statistics toolbox of MATLAB [52]. Metabolite concentrations were normalised by dividing each value by their mean value over all 65 spectra. The MANOVA was performed as a discriminate function analysis to generate canonical variables which had the maximal separation of the means of the 6 different tumour types. Each canonical variable is a linear combination of the normalised concentration values of the 29 different metabolites.

## Abbreviations used

3T: 3 Tesla; Ace: Acetate; Ala: Alanine; ANOVA: Analysis of Variance; Asc: Ascorbate; Asp: Aspartate; Bet: Betaine; Cho: Choline; CPMG: Carr-Purcell-Meiboom-Gill; Cr: Creatine; GBM: Glioblastoma Multiforme; Glc: Glucose; Glu: Glutamate; Gln: Glutamine; Glx: Glutamate plus Glutamine; GSH: Glutathione; GPC: Glycerophospho-choline; GPE: Glycerophospho-ethanolamine; Gly: Glycine; His: Histidine; hr: hour; HRMAS: High Resolution Magic Angle Spinning; H-Tau: Hypo-taurine; Ile: Isoleucine; Lac: Lactate; Leu: Leucine; Lys: Lysine; MANOVA: Multi-variate Analysis of Variance; min: minute; M-Ins: Myo-Inositol; MRS: Magnetic Resonance Spectroscopy; ms: milli-second; NAA: N-acetyl-aspartate; NMR: Nuclear Magnetic Resonance; PCh: Phosphocholine; PE: Phospho-ethanolamine; ppm: part-per-million; S-Ins: Scyllo-inositol; Suc: Succinate; Tau: Taurine; Thr: Threonine; TR: Repetition time; TTA: Total Time of Acquisition; TOCSY: Total Correlation Spectroscopy; Val: Valine.

## Competing interests

The authors declare that they have no competing interests.

## Authors' contributions

AJW designed and performed the HRMAS experiments and their analysis and drafted the paper. GF collected the tumour samples and helped draft the manuscript. JRG and BAB participated in the design and coordination of the study and helped draft the manuscript. MW provided the simulation software and helped draft the manuscript. FAH conceived of the study, participated in its design and coordination and helped to draft the manuscript. All authors read and approved the final manuscript.
